# Physiological effects of environmentally relevant, multi-day thermal stress on wild juvenile Atlantic salmon (*Salmo salar*)

**DOI:** 10.1093/conphys/cox014

**Published:** 2017-02-27

**Authors:** Emily Corey, Tommi Linnansaari, Richard A. Cunjak, Suzanne Currie

**Affiliations:** 1 Department of Biology and Canadian Rivers Institute, University of New Brunswick, P.O. Box 4400, Fredericton, New Brunswick, CanadaE3B 5A3; 2 Department of Biology, Mount Allison University, 63B York, Street, Sackville, New Brunswick, Canada E4L 1G7

## Abstract

The frequency of extreme thermal events in temperate freshwater systems is expected to increase alongside global surface temperature. The Miramichi River, located in eastern Canada, is a prominent Atlantic salmon (*Salmo salar*) river where water temperatures can exceed the proposed upper thermal limit for the species (~27°C). Current legislation closes the river to recreational angling when water temperatures exceed 20°C for two consecutive nights. We aimed to examine how natural thermal variation, representative of extreme high thermal events, affected the thermal tolerance and physiology of wild, juvenile Atlantic salmon. We acclimated fish to four thermal cycles, characteristic of real-world thermal conditions while varying daily thermal minima (16°C, 18°C, 20°C or 22°C) and diel thermal fluctuation (e.g. Δ5°C–Δ9°C). In each cycling condition, we assessed the role that thermal minima played on the acute thermal tolerance (critical thermal maximum, (CTMax)), physiological (e.g. heat shock protein 70 (HSP70), ubiquitin) and energetic (e.g. hepatic glycogen, blood glucose and lactate) status of juvenile Atlantic salmon throughout repeated thermal cycles. Exposure to 16–21°C significantly increased CTMax (+0.9°C) compared to a stable acclimation temperature (16°C), as did exposure to diel thermal fluctuations of 18–27°C, 20–27°C and 22–27°C, yet repeated exposure provided no further increases in acute thermal tolerance. In comparison to the reference condition (16–21°C), consecutive days of high temperature cycling with different thermal minima resulted in significant increases in HSP70 and ubiquitin, a significant decrease in liver glycogen, and no significant cumulative effect on either blood glucose or lactate. However, comparison between thermally taxed treatments suggested the diel thermal minima had little influence on the physiological or energetic response of juvenile salmon, despite the variable thermal cycling condition. Our results suggest that relatively cooler night temperatures in the summer months may play a limited role in mitigating physiological stress throughout warm diel cycle events.

## Teaser

River temperatures often surpass the thermal limits of juvenile Atlantic salmon (*Salmo salar*). Using thermal monitoring data to replicate a natural heat event, we investigated how cooler nighttime temperatures would affect how juvenile salmon cope with several days of heat cycling.

## Introduction

Climate warming is expected to alter distribution patterns of fishes, with temperatures in some systems already approaching the upper thermal tolerance of endemic species (Caissie, 2006). In salmonids, warming water temperatures affects migration (Crossin *et al*., 2008), smoltification (McCormick *et al*., 1999), growth and survival (Swansburg *et al*., 2002). The Miramichi River, located in eastern Canada, is a prominent Atlantic salmon (*Salmo salar*) river (DFO, 2013) where maximum summer temperatures can reach 27–30°C (Caissie *et al*., 2014), well beyond the range for optimal growth in juveniles (~15–20°C; Jobling, 1981; Elliott and Hurley, 1997; Jonsson *et al*., 2001; Elliott and Elliott, 2010). The upper threshold for normal feeding behavior (i.e. 22–24°C; Breau *et al*., 2011) can be surpassed for as many as 62 days during the spring/summer (Caissie *et al*., 2012). Peak water temperatures typically occur in conjunction with diel fluctuations of ≤9°C in summer months (our unpublished work). Thus, the Miramichi River is an ideal system for understanding the effects of climate warming on salmon physiology. An appreciation of a fish's physiological capacity to cope with climate-driven environmental stress is emerging as a powerful approach in conservation and management as has been demonstrated for Pacific salmon (Cooke *et al*., 2012, Muñoz *et al*., 2014).

Natural diel thermal cycles impact thermal tolerance (Wehrly *et al*., 2007), bioenergetics (Beauregard *et al*., 2013; Eldridge *et al*., 2015), and metabolic status (Oligny-Hébert *et al*., 2015) of fishes. However, largely due to practical issues of temperature control in the laboratory, comparatively few studies investigate how temperature cycling, compared to stable acclimation temperatures, affect fish biology (see Threader and Houston, 1983; Houston and Gingras-Bedard, 1994; Mesa *et al*., 2002; Narum *et al*., 2013; Tunnah *et al*., 2017 as examples). Given that water temperatures in traditionally productive Atlantic salmon habitat, such as the Miramichi River, are increasing beyond the presumed thermal limit for this species (Elliott and Elliott, 1995), we were interested in whether the nature of the thermal cycle differentially influenced physiology. Specific to current federal (DFO) regulations whereby river closures occur when water temperature is ≥20°C for two consecutive nights (DFO, 2012), we investigated the effects of warming temperature cycles with distinct thermal minimums on the physiological response (i.e. thermal tolerance) of juvenile salmon. Although federal guidelines are designed primarily with mature life stages in mind, juveniles were used as a readily accessible and abundant proxy for their adult counterpart. The link between adult and juvenile fish physiology has been defined in many fishes (see Rodnick *et al*., 2004; Pörtner *et al*., 2008; Fowler *et al*., 2009; Morita *et al*., 2010), and although a more thermally tolerant life stage, juveniles provide insight into the physiological capacity of salmonids to withstand extreme events.

Our main objective was to determine how distinct, natural thermal variation, representative of summer high temperature events, affected the thermal tolerance and physiology of wild juvenile Atlantic salmon, with the goal of informing conservation and management efforts. To this end, we acclimated fish to four thermal cycles, representative of real-world conditions but with differing minimum nighttime temperatures (16°C, 18°C, 20°C, 22°C) and diel temperature fluctuations (e.g. Δ5°C–Δ9°C). In each cycling condition, we measured critical thermal maximum (CTMax) as a proxy for acute thermal tolerance (Beitinger *et al*., 2000), recognizing that CTMax is influenced by acclimation temperature (e.g. Fangue *et al*., 2006). We also measured indicators of physiological (HSP70, ubiquitin) and energetic (lactate, glycogen) stress. We hypothesized that nighttime temperature throughout a thermal event dictates physiological limits. If supported, then we predicted that fish exposed to conditions with the highest nighttime temperature will have reduced acute thermal tolerance, and will experience enhanced metabolic and cellular stress compared to those exposed to cooler nighttime temperatures.

## Methods

### Fish collection & maintenance

Wild Atlantic salmon parr (N = 320; fork length = 6.1–10.4 cm; weight = 1.9–12 g) were collected on 16 June 2013, using a Smith-Root LR-24 backpack electrofisher from the Cains River (N46°25′59. 5; W066°01′29. 1—N46°26′05. 0; W066°01′10. 8 ± 15 m), a tributary of the Southwest Miramichi River. Fish were transported to the Harold Crabtree Aquatic Facility at Mount Allison University, NB, Canada and placed in a 750 L (circular fiberglass) recirculation holding tank at 15.00 ± 0.03°C. Fish were fed a mixture of dehydrated krill and commercial pellet feed (Corey Nutrition Company) twice daily until satiation. Water temperature was maintained until parr were weaned to feed exclusively on pellets (~10 days). Fish were subsequently fed once daily to satiation and exposed to a fluctuating ‘acclimation regime’ with nighttime minimum and daytime maximum temperatures (*T*_min_ and *T*_max_) of 16 and 21 ± 0.2°C, respectively (mean ± SD; 12 h warming: 12 h cooling) with a natural photoperiod (~16 h light:8 h dark). Water temperature was monitored using an iBCod temperature logger (±1°C, 15 min interval, Alpha Mach Inc.) and dissolved oxygen (DO) was measured daily (7.5–10 mg L^−1^; YSI Pro 20, Xylem Inc.).

### Experimental design

**Figure 1:**
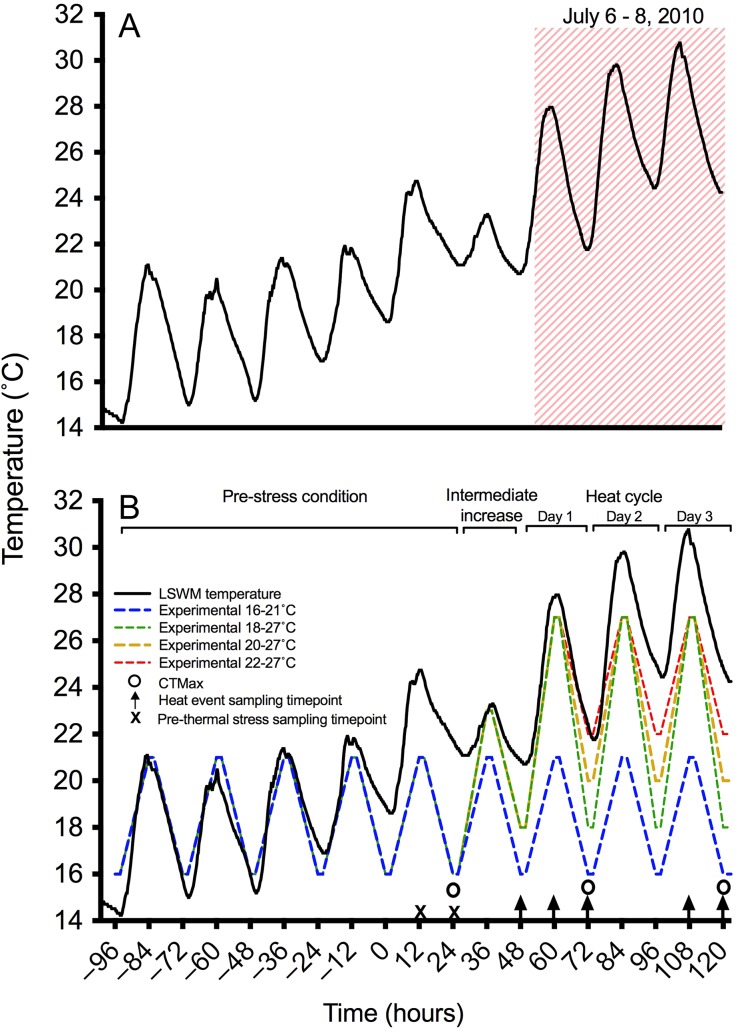
(**A**) Thermal profile of the Little Southwest Miramichi (LSWM) River throughout early July 2010. A known thermal event occurred July 6–8. (**B**) Thermal profile of the LSWM River overlaid with experimental treatments. Details provided in text of Methods.

A significant heat event occurred in the Little Southwest Miramichi River (LSWM) in early July 2010 (Fig. 1A). Maximum daytime water temperatures during the July 6–8 event ranged from 28.0°C to 30.7°C (mean: 29.3 ± 0.5°C); nighttime temperatures ranged from 20.7°C to 24.5°C (mean: 22.8 ± 0.9°C). Such temperatures are known to alter metabolism in juvenile Atlantic salmon (Breau *et al*., 2011). To address our objectives, we chose to model this particular heat event for our experimental treatments. We established an acclimation regime based on the mean diel *T*_max_ and *T*_min_ of the 2 weeks prior to the event (16–21°C). To minimize thermal stress mortality, *T*_max_ was set at 27°C for all treatments, just below the upper incipient lethal limit (7-day survival: 27.8°C; Elliott, 1991). Three temperature treatments were established within the limits of Δ*T* measured in the LSWM River in 2010 (Fig. 1B). We established minimum (i.e. nighttime) water temperatures as: (i) above the current legislated diel minimum temperature that determines river closure (≥20°C; DFO, 2012), 22–27°C; (ii) at the threshold, 20–27°C; and (iii) within the optimum range for growth and survival, 18–27°C. In order to subject parr to 27°C without exceeding the maximum natural diel Δ*T* (9°C) measured in the LSWM, an intermediate (‘ramp’) day was placed between the 16–21°C-acclimation regime and the temperature treatments. Temperatures on this day reached a maximum of 23°C and minimum of 18°C (Fig. 1B).

## Series 1: Thermal physiology

### Diel thermal cycles and sampling

Once in experimental tanks, fish remained at 16–21°C for a 5-day acclimation period. All fish were then subjected to a 1-day intermediate exposure (Day-6; 18–23°C) prior to exposure to 3 days of heat cycling (Day 7–9; *T*_max_ = 27°C in all treatments) with treatment-specific thermal minima. A fourth treatment group was maintained at 16–21°C throughout the experimental procedure. We ran two experimental trials, each lasting 9.5 days (Trial 1: 16–21°C and 22–27°C treatments, July 13–22; Trial 2: 18–27°C and 20–27°C treatments, July 22–31). Each thermal treatment was assigned a bank of three 300 L circular fiberglass tanks with each bank connected to separate recirculation systems. Parr were randomly selected from the holding tank (at 16–21°C) and transferred to one of the two tank banks (i.e. total *N =* 6 tanks). Fish were distributed equally among all experimental tanks (i.e. 108 fish per trial; ~36 fish per tank). For each sampling event, nine fish were sampled from a single tank within a bank. Sampling regimens were scheduled such that no tank was subject to consecutive samplings in order to eliminate recurring stress from repeated sampling within short time periods (minimum time between sampling a particular tank = 48 h). Fish were not fed 24 h prior to sampling.

Fish were sampled throughout the diel temperature cycle according to the following schedule: (i) *T*_min_ following the ‘intermediate’ temperature increase (*t* = 48 h); (ii) *T*_max_, the first day of heat cycling (*t* = 60 h); (iii) the first *T*_min_ throughout heat cycle (16°C, 18°C, 20°C or 22°C, depending on treatment, *t* = 72 h); (iv) *T*_max_, the third day of heat cycling (*t* = 108 h); and (v) *T*_min_, the third day of heat cycling (*t* = 120 h, Fig. 1B). These sampling time points were chosen to establish the physiological condition prior to heat exposure, the implications of 1 day of heat cycling, and the potential cumulative effect of 3 days of cycling. This 5-timepoint sampling regime was performed on all treatment groups. However, to address the potential effect of ‘trial’ on our results, two additional sampling time points (*t* = 12 h and *t* = 24 h) were established in one treatment group in each of the two trials (16–21°C for Trial 1 and 18–27°C for Trial 2), prior to the heat cycle. This additional sampling allowed us to further address the incipient, ‘pre-thermal stress’ condition.

Fish were anaesthetized with a buffered ethyl 3-aminobenzoate methanesulfonate (MS-222; Sigma-Aldrich) solution with supplemental aeration. Once anesthetized, mass and fork length were measured, and blood extracted from the caudal artery. Whole blood (WB) lactate and glucose were measured using a Lactate Pro™ Portable Blood Lactate Analyzer and a OneTouch® Ultra 2 Meter (Gallant *et al*., 2017). Environmental stressors are known to alter the blood chemistry of fish. Blood glucose levels rise to supply ATP and fuel activity if the situation becomes critical. Similarly, blood lactate is a byproduct of anaerobic metabolism, and an increase in this metabolite indicates a switch to anaerobic ATP production suggesting aerobic energy stores are diminished. Parr were terminated by a swift blow to the head followed by severance of the spinal cord. Liver was dissected, immediately frozen in liquid nitrogen and stored at −80°C until processing. Blood samples were centrifuged at 5000 rpm for 3 min and 13°C to separate blood constituents. White blood cells and plasma were discarded while red blood cells (RBC) were flash frozen in liquid nitrogen and stored at −80°C.

## Series 2: Critical thermal maximum

CTMax tests were performed on 9–11 salmon parr at each of three *T*_min_ time points (between 04:00 and 09:00); to establish an initial pre-thermal stress value (24 h), the effect of a singular thermal cycle (60 h), and of multiple heat cycles (120 h; Fig. 1B). Prior to testing, fish were transported to a plastic CTMax experimental chamber (40 × 13.5 × 11 cm) with a clear Plexiglas® lid. Chambers contained an aerated water flow of 4 L min^−1^ and had an initial water temperature matching that of the experimental procedure. Once the fish was in the chamber, water temperature was increased acutely at a rate 0.32 ± 0.002°C min^−1^ (mean ± SEM; Becker and Genoway, 1979) until fish lost equilibrium. DO and temperature were measured at 1-min intervals; DO saturation remained >72% in all trials.

In addition to protocol described above, a separate group of 10 parr were isolated at the time of field collection and maintained at a constant 16°C for 1 week prior to being measured for fork length (±1 mm), wet weight (±0.1 g) and individually tagged with visible implant elastomer (Northwest Marine Technology Inc.). Fish recovered for 60 h prior to CTMax testing on 4 July 2013 at 06:00 h. After testing, fish recovered at 16°C for 5 days prior to acclimation to 16–21°C for 36 days. The test was then repeated under the same conditions to evaluate the influence of the thermal cycle on CTMax.

## Analyses

### Glycogen

Protocols for the liver glycogen extraction (Clow *et al*., 2004) and assay were modified from Bergmeyer *et al*. (1974). Hydrolysates were frozen at −80°C until analyzed for glucose content. The glucose assay was modified to use 25 µl of sample, diluted 1:7 in assay media (Clow *et al*., 2004) and added to each well of a microtiter plate. Twenty-five microlitres of 100 µl/ml G-6-PDH was added to each well to eliminate any endogenous G-6-P that remained in solution. The plate was read on a VERSAmax Tunable Microplate Reader (Molecular Devices Corporation) at 340 nm until absorbance stabilized. Hexokinase (25 µl) was then added and the absorbance was read after 15–25 min.

### Heat shock protein 70

The induction of heat shock protein 70 (HSP70) may be considered an ecologically relevant indicator of thermal stress for Atlantic salmon (Lund *et al*. 2002; Chadwick *et al*., 2015; Tunnah *et al*., 2017). Soluble protein in liver and RBC were extracted as in Tunnah *et al*. (2017) and LeBlanc *et al*. (2011), respectively, and assayed using the Lowry-based DC protein assay kit (Bio-Rad, Mississauga, ON, Canada). Standards (bovine serum albumin; Bio-Rad) and samples were diluted in protein extraction buffer or salmon saline, respectively, and absorbance read at 750 nm using a SpectraMax M5 plate reader and SoftMax Pro software. Samples were prepared using 15 µg soluble protein and western blots performed as in Kolhatkar *et al*. (2014). We used a rabbit anti-salmonid HSP70 primary antibody (AgriSera, AS05061; 1:50 000 dilution in blocking buffer) and a goat anti-rabbit secondary (Enzo Life Sciences; SAB-300 1:50 000) for immunodetection. This antibody is specific for the inducible form of HSP70 and does not cross-react with the constitutive isoform in salmonids (Rendell *et al*., 2006; Fowler *et al*., 2009; Tunnah *et al*., 2017). Protein bands were visualized using an ECL Advance Chemiluminescent Western Blotting Detection kit (Amersham Pharmacia Biotech) imaged using a Versadoc Imaging System and Quantity One software (Bio-Rad). Relative band density was quantified using Image Lab software (Bio-Rad) and calculated from the standard curve on each blot.

### Ubiquitin

We used ubiquitin (Ub) as an indirect measure of protein damage/turnover. Soluble protein samples of known concentration were diluted and 5 µg of protein was dotted on a nitrocellulose membrane as in MacLellan *et al*. (2015). To ensure equal protein loading, a Ponceau-S stain (Sigma-Aldrich) was applied to each membrane after imaging, according to manufacturer's instructions.

## Calculations and statistical analyses

Data were analyzed using R statistical software (R Development Core Team, 2016). Prior to analyses, data were divided into two sections: *t* = 12 h and *t* = 24 h to assess the effect of ‘trial’ (i.e. temperature regime shared by all experimental groups), and *t* = 48 h through *t* = 120 h to test experimental effects of temperature and time. In the case of glycogen, WB glucose, WB lactate, HSP70 (liver & RBC) and liver Ub, data were log-transformed to meet the assumptions of normality and homoscedasticity. A linear model two-way ANOVA was conducted to compare the main effects of independent variables (thermal cycle and time point) and the inherent interaction effect on indicators of physiological and energetic stress. In case of a significant interaction between sampling time point and thermal cycle, a one-way ANOVA was used to discern differences between temperature treatments at individual time points. Pre-thermal stress sampling time points were treated in a similar fashion with the exception of the CTMax data, as only one pre-thermal stress time point occurred. In this instance, a linear one-way ANOVA was used to test for the effect of trial. When appropriate, a subsequent Tukey *post-hoc* test was used. In all cases, *α* = 0.05 and values were expressed as mean ± SEM. Although our study does not use a repeated measures design, line graphs are presented for a clearer view of trends within the data.

## Results

### Pre-thermal stress sampling

Pre-thermal stress sampling time points differed in three of the variables analyzed (Table 1). There was a significant effect of sampling time (*F*_1, 1_ = 8.45, *P =* 0.006) and thermal cycle (*F*_1, 1_ = 6.70, *P* = 0.014) with no significant interaction (*F*_1, 1_ = 1.23, *P =* 0.276) in WB glucose. Significant differences occurred in incipient pre-exposure values for liver HSP70, where there was an effect of sampling time (*F*_1, 1_ = 27.62, *P =* <0.001) and thermal cycle (*F*_1, 1_ = 8.28, *P* = 0.008), but not the interaction (*F*_1, 1_ = 0.28, *P* = 0.601). Significant differences were also observed in RBC HSP70 where there was an effect of sampling time (*F*_1, 1_ = 16.88, *P* = < 0.001) and thermal cycle (*F*_1, 1_ = 182.09, *P* = <0.001), but not the interaction (*F*_1, 1_ = 0.06, *P =* 0.11; Table 1). No significant effects of sampling time or thermal cycle were observed in CTMax, WB lactate, liver Ub or RBC Ub (see Table 1).

**Table 1:** Two-way ANOVA performed on physiological variables of juvenile Atlantic salmon exposed to pre**-**thermal stress sampling conditions (16–21°C)

Endpoint	Time			Treatment			Interaction		
	df	F-statistic	P-value	df	F-statistic	P-value	df	F-statistic	P-value
CTMax**	–	–	–	1	4.07	0.073	–	–	–
L glycogen	1	1.26	0.272	1	0.99	0.330	1	0.57	0.458
WB glucose	1	8.45	0.006*	1	6.70	0.014*	1	1.23	0.276
WB lactate	1	0.07	0.790	1	0.15	0.702	1	2.79	0.106
L HSP70	1	27.62	<0.001*	1	8.28	0.008*	1	0.28	0.601
L Ub	1	0.23	0.633	1	0.22	0.640	1	0.30	0.588
RBC HSP70	1	16.88	<0.001*	1	182.09	<0.001*	1	2.67	0.113
RBC Ub	1	0.12	0.737	1	0.28	0.598	1	0.35	0.558

CTMax = critical thermal maximum; L = liver; M = muscle; WB = whole blood; RBC = red blood cell; HSP70 = heat shock protein 70; Ub = ubiquitin. Asterisks indicate significance of two-way ANOVA with α = 0.05 and P <0.05.

**One-way ANOVA performed.

### Critical thermal maximum

**Figure 2:**
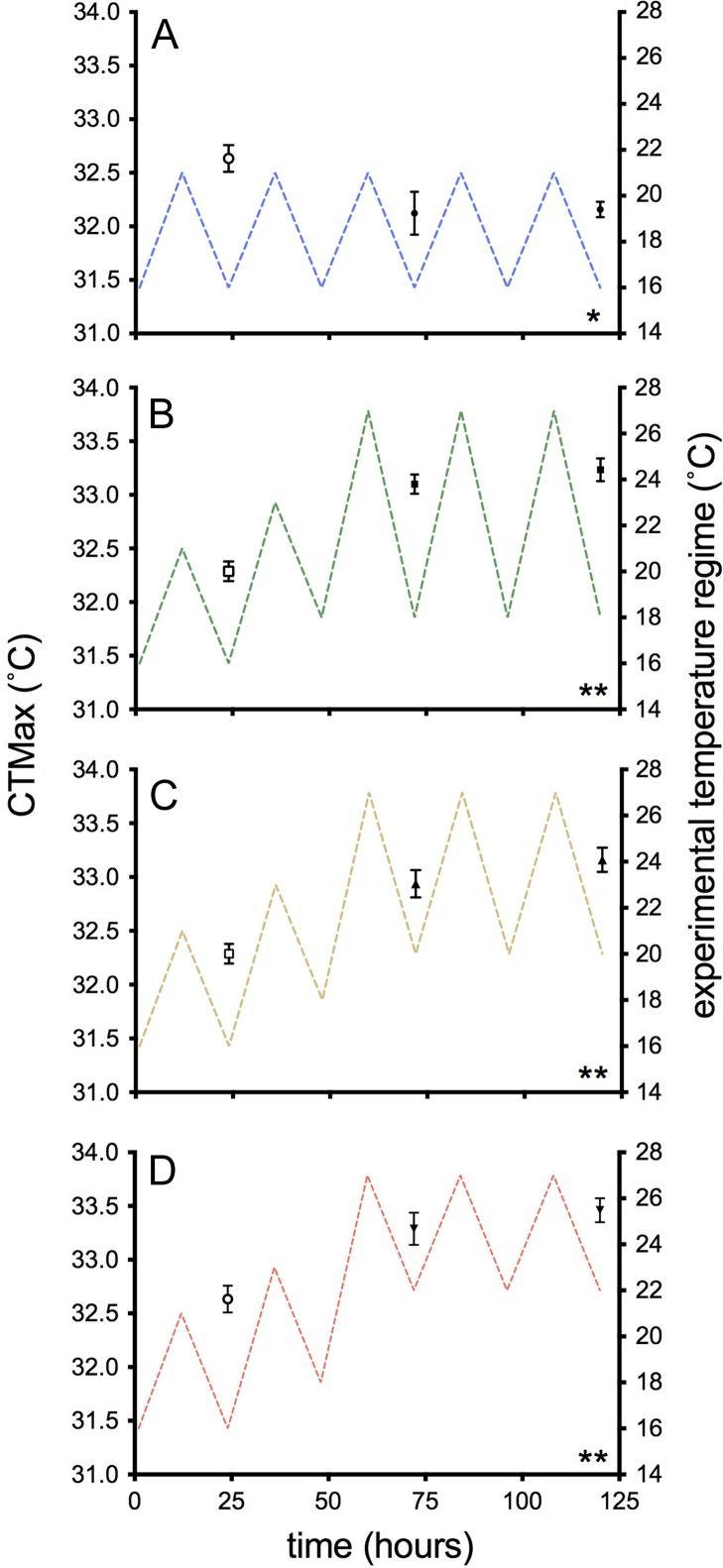
CTMax of wild juvenile salmon exposed to diel cycles: (**A**) 16–21°C (control group); or multi-day thermal stress (closed symbols; 60–120 h) of (**B**) 18–27°C; (**C**) 20–27°C; (**D**) 22–27°C. Data are presented as mean ± SEM (*n* = 5–10). Open symbols (*t* = 24 h) represent pre-thermal stress sampling time points and were secondarily used to assess the effect of ‘trial’. Asterisks indicate significant differences among treatments (*P* < 0.05).

Fish exposed to 16–21°C lost equilibrium at 32.5 ± 0.09°C, a temperature significantly higher than those maintained at a stable acclimation temperature of 16°C (31.4 ± 0.09°C; *F*_2, 48_ = 43.0, *P <* 0.001). Overall, CTMax increased in all thermally cycled groups compared to the 16–21°C-control group (*F*_3, 64_ = 31.92, *P <* 0.001). Significant increases in CTMax were observed in all temperature treatments after one day of cycling (*t* = 72 h; *P <* 0.001), with no significant differences observed between groups (Fig. 2B–D; *P* = 0.11–0.77). No significant differences were observed between *t* = 72 h and *t* = 120 h of heat cycling within or between temperature treatments (pooled mean of all heat exposed fish = 33.1 ± 0.08°C; *P* = 0.96–0.99; Table 2; Fig. 2).

**Table 2:** Two-way ANOVA performed on physiological variables of juvenile Atlantic salmon exposed to multi-day thermal stress

Endpoint	Time			Treatment			Interaction		
	df	F-statistic	P-value	df	F-statistic	P-value	df	F-statistic	P-value
CTMax	1	1.14	0.290	3	31.93	<0.001*	3	0.16	0.920
L glycogen	4	6.93	<0.001*	3	6.73	<0.001*	12	2.63	0.003*
WB glucose	4	1.72	0.149	3	2.55	0.057	12	1.44	0.155
WB lactate	4	5.65	<0.001*	3	2.33	0.077	12	1.74	0.062
L HSP70	4	100.61	<0.001*	3	177.05	<0.001*	12	9.68	<0.001*
L Ub	4	4.29	0.002*	3	2.54	0.058	12	1.38	0.179
RBC HSP70	4	73.18	<0.001*	3	207.50	<0.001*	12	22.25	<0.001*
RBC Ub	4	4.78	0.001*	3	15.03	<0.001*	12	1.07	0.388

Asterisks indicate significance of two-way ANOVA with α = 0.05 and P <0.05.

### Metabolites

**Figure 3:**
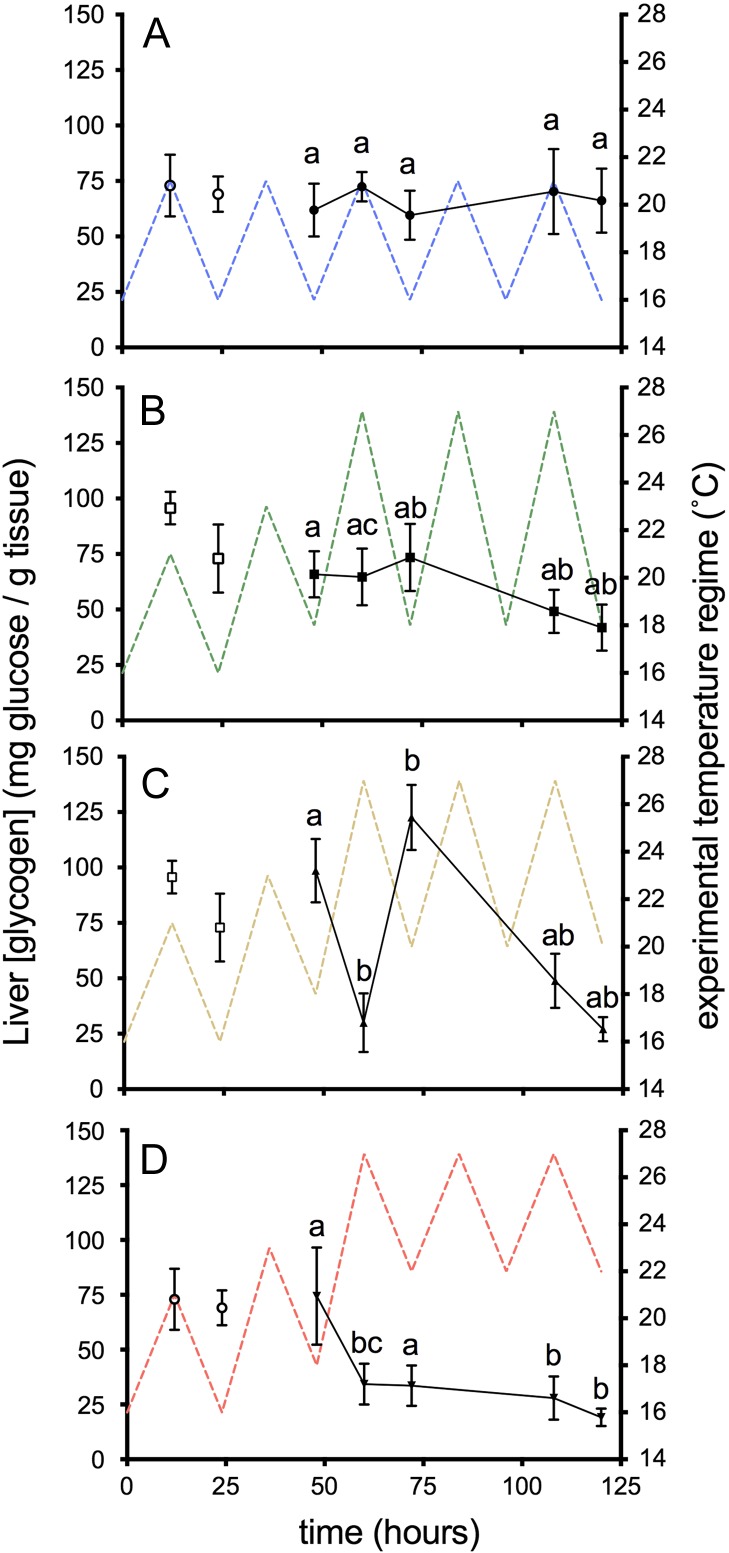
Liver glycogen in juvenile salmon exposed to diel cycles: (**A**) 16–21°C (control group); or multi-day thermal stress (60–120 h) of (**B**) 18–27°C; (**C**) 20–27°C; (**D**) 22–27°C. Data are presented as mean ± SEM (*n* = 6–9). Pre-thermal stress sampling (*t* = 12 and 24 h) assessed the effect of ‘trial’ and was not included in the analysis. Letters indicate significant differences among treatments (*P* < 0.05) within sampling time points.

Liver glycogen levels in the 16–21°C group remained relatively stable at a mean of 67.4 ± 4.8 mg g^−1^ throughout the experimental sampling period (Fig. 3). Although liver glycogen decreased over time in all but the 16–21°C group, a significant interaction was observed between time and temperature treatment (*F*_12, 160_ = 2.63, *P <* 0.003; Table 2) indicating that the pattern of decline was different in each thermally cycled group. No significant differences in glycogen were observed prior to the first heat cycle in any temperature treatment (*t* = 48 h; *F*_3, 27_ = 1.08, *P* = 0.37; Fig. 3). Differences among thermally cycled groups were apparent at the peak of the first heat cycle (*t* = 60 h). The 20–27°C and 22–27°C groups experienced the most pronounced decline in liver glycogen, but by the end of the experiment (*t* = 120 h) only the 22–27°C group was significantly different than the 16–21°C group (19.1 ± 4.0 mg·g^−1^, *P* = 0.006). One noteworthy exception to the overall pattern is the observed spike in liver glycogen at *t* = 72 h in the 20–27°C group (Fig. 3C) that was significantly greater than the 16–21°C and 22–27°C groups (*P* = 0.04 and *P <* 0.001, respectively).

WB glucose did not change with temperature treatment (*F*_3, 160_ = 2.56, *P* = 0.057) or time (*F*_4, 160_ = 1.72, *P* = 0.15; Table 3). However, as noted above, significant differences were observed between WB glucose pre-thermal stress time points (*P* = 0.007; Table 1).

**Table 3:** Mean whole blood lactate and glucose (±SEM) in juvenile Atlantic salmon exposed to 16–21°C (control group) for 5 days; or multi-day thermal stress (18–27°C, 20–27°C or 22–27°C)

Treatment (°C)	Time point (h)						
	12^*^	24^**^	48^a^	60^a^	72^a^	108^a^	120^a^
Whole blood glucose							
16–21	1.9 ± 0.29	3.2 ± 0.37	3.1 ± 0.32	2.1 ± 0.39	2.1 ± 0.37	2.0 ± 0.27	2.0 ± 0.19
18–27	1.5 ± 0.15	2.0 ± 0.26	2.9 ± 0.29	2.7 ± 0.56	2.0 ± 0.26	2.7 ± 0.36	1.8 ± 0.18
20–27	–	–	1.8 ± 0.26	3.2 ± 0.42	2.7 ± 0.15	3.0 ± 0.42	1.9 ± 0.31
22–27	–	–	2.9 ± 0.28	2.8 ± 0.64	2.1 ± 0.36	1.9 ± 0.30	2.3 ± 0.28

	12^*^	24^*^	48^a^	60^b^	72^ab^	108^b^	120^a^
Whole blood lactate							
16–21	2.6 ± 0.28	3.3 ± 0.42	3.7 ± 0.44	3.4 ± 0.60	4.6 ± 0.71	4.8 ± 0.85	2.1 ± 0.22
18–27	3.7 ± 0.83	2.8 ± 0.34	3.1 ± 0.39	4.5 ± 0.88	2.6 ± 0.25	3.8 ± 0.30	1.9 ± 0.39
20–27	–	–	2.5 ± 0.37	4.3 ± 0.42	2.9 ± 0.37	4.5 ± 0.50	4.0 ± 0.73
22–27	–	–	3.6 ± 0.79	5.2 ± 0.50	3.2 ± 0.36	4.8 ± 0.92	3.2 ± 0.16

Stress occurred from 60 to 120 h. Pre-thermal stress sampling (12 and 24 h) assessed the effect of ‘trial’ and ‘bank’ and was analyzed separately.

Letters are indicative of significance between sampling time points during thermal ramping (*t* = 48 h–120 h; *P* < 0.05). Asterisks are indicative of significance between pre**-**thermal stress sampling time points.

We did not observe any differences in blood lactate among temperature treatments (*F*_3, 160_ = 2.33, *P* = 0.08; Table 3); however, we did note differences in blood lactate over time (*F*_4, 160_ = 5.65, *P <* 0.001). Blood lactate at *T*_min_ throughout the elevated thermal cycles (*t* = 72–120 h) did not differ from the pre-thermal stress intermediate *T*_min_ (*t* = 48; *P* = 0.96 and *P* = 0.87, respectively), nor was there a significant difference at the peak of the first heat cycle (*t* = 60 h; *P* = 0.06). However, blood lactate was significantly higher at the peak of the third heat cycle compared with the initial pre-temperature stress condition prior to thermal ramping (*t* = 108 h; *P* = 0.02). Significant decreases in blood lactate were observed at *t* = 120 h compared with both thermal peaks (*t* = 60 h and *t* = 108 h; *P* = 0.004 and *P* = 0.001, respectively).

### Cellular stress response

**Figure 4:**
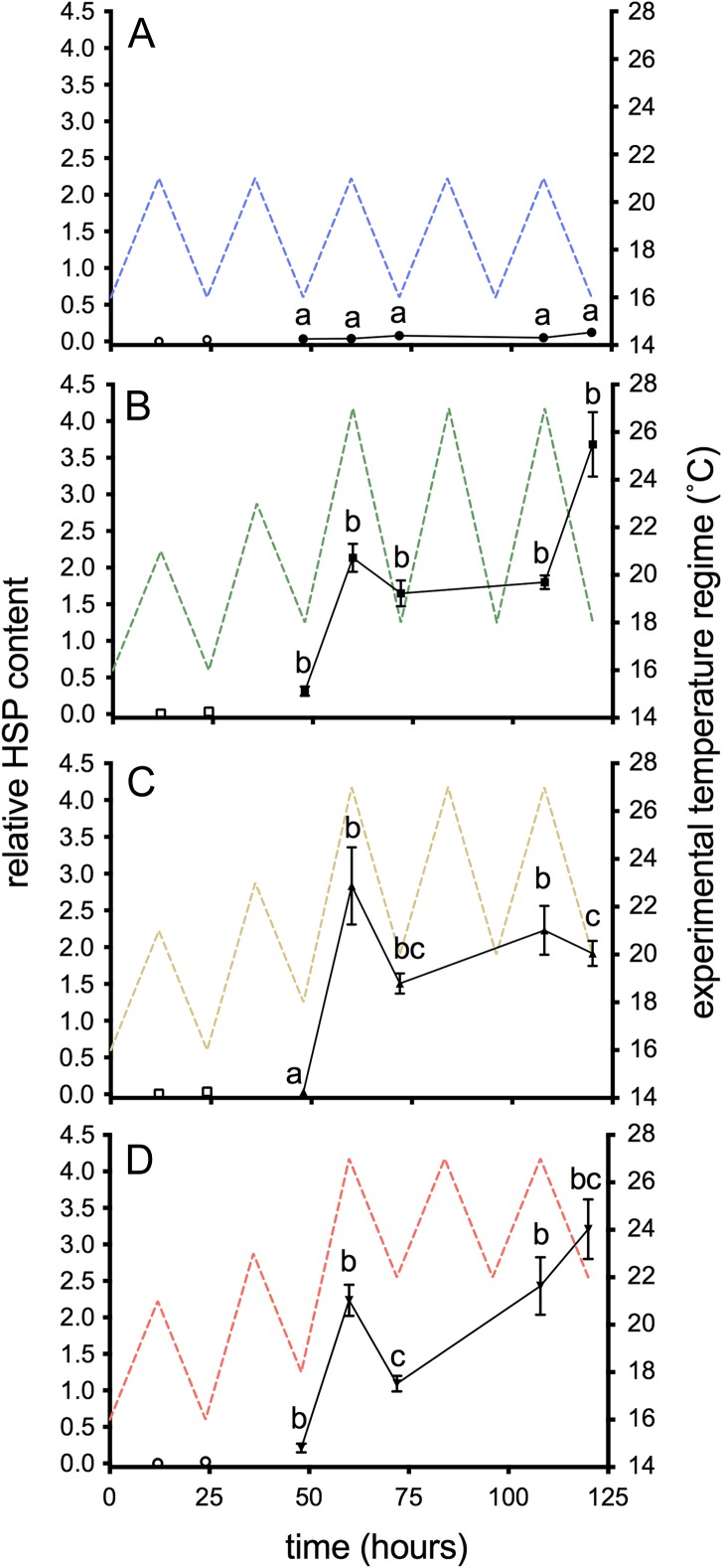
Liver HSP70 in juvenile salmon exposed to diel cycles: (**A**) 16–21°C only; or multi-day thermal stress (closed symbols; 60–120 h) of (**B**) 18–27°C; (**C**) 20–27°C; (**D**) 22–27°C. Data are presented as mean ± SEM (*n* = 5–9). Pre-thermal stress sampling (open symbols; *t* = 12 & 24 h) assessed the effect of ‘trial’ and was not included in the analysis. Letters indicate significant differences among treatments (*P* < 0.05) within sampling time points.

Liver HSP70 was not induced under control conditions (Fig. 4A); however, a significant interaction was observed with time and temperature treatment (*F*_12, 160_ = 9.68, *P <* 0.001), indicating that the pattern of induction depended on thermal regime. Liver HSP70 was induced in the 18–27°C and 22–27°C groups at the pre-thermal stress sampling point after exposure to 23°C (*t* = 48 h; *P <* 0.001 & *P* = 0.02, respectively). The peak of the first day of heat exposure induced a significant increase in liver HSP70 in all three high thermal cycles compared to the 16–21°C group (*t* = 60 h; *P <* 0.001; Fig. 4); however, HSP70 was not significantly different among the temperature treatments. Although the specific pattern of liver HSP70 induction varied among temperature treatments, levels remained elevated throughout the duration of the 3-day heat event (*t* = 60–120 h; *P <* 0.001 in all cases). After the onset of the event, treatments did not vary from one another statistically (*P* = 0.07–0.99), with the exceptions of the diel minima following the first and third heat cycles (*t* = 72 and 120 h) where HSP70 levels in the 18–27°C were significantly greater than in the 22–27°C group (*P* = 0.018 and 0.008, respectively; Fig. 4).

**Figure 5:**
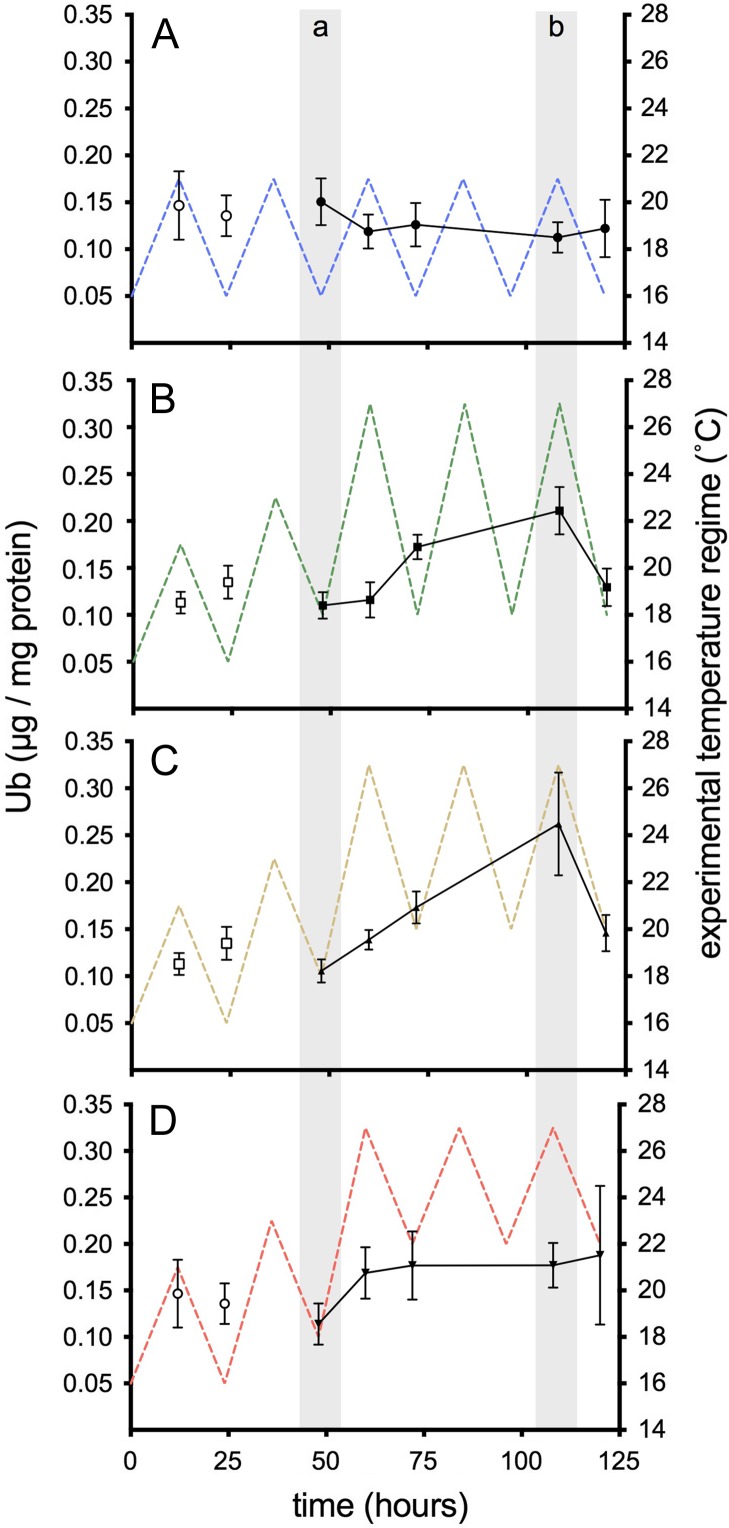
Liver Ub in juvenile salmon exposed to diel cycles: (**A**) 16–21°C only; or multi-day thermal stress (closed symbols; 60–120 h) of (**B**) 18–27°C; (**C**) 20–27°C; (**D**) 22–27°C. Data are presented as mean ± SEM (*n* = 6–9). Pre-thermal stress sampling (open symbols; *t* = 12 and 24 h) assessed the effect of ‘trial’ and was not included in the analysis. Lettered shaded bars indicate significant differences between time points (*P* < 0.05).

As was the case with HSP70, liver Ub was significantly increased over time (*F*_4, 160_ = 4.29, *P* = 0.002; Fig. 5), but we did not observe statistically significant differences among thermal groups (*F*_3, 160_ = 2.54, *P* = 0.058; Table 2). Although in several cases, differences between sampling time points approached significance (*t* = 48 h and *t* = 72 h, *P* = 0.06; *t* = 60 h and *t* = 108 h, *P* = 0.07; *t =* 108 and *t* = 120 h, *P* = 0.066), the only significant difference in liver Ub was observed at *t* = 108 h, where Ub was significantly higher than pre-thermal exposure values, *t* = 48 h (*P* = 0.003).

**Figure 6:**
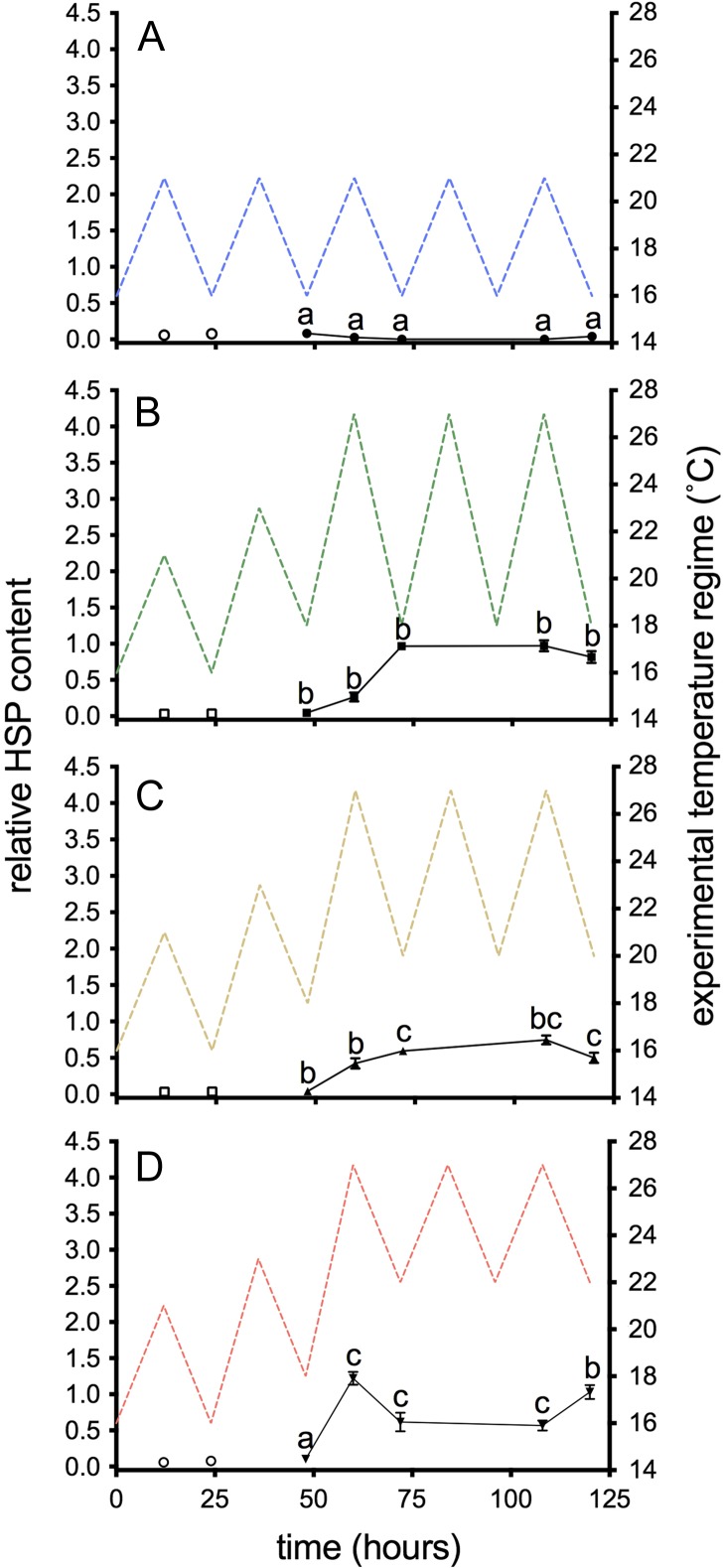
RBC HSP70 in juvenile salmon exposed to diel cycles: (**A**) 16–21°C only; or multi-day thermal stress (closed symbols; 60–120 h) of (**B**) 18–27°C; (**C**) 20–27°C; (**D**) 22–27°C. Data are presented as mean ± SEM (*n* = 5–9). Pre-thermal stress sampling (open symbols; *t* = 12 and 24 h) assessed the effect of ‘trial’ and was not included in the analysis. Letters indicate significant differences among treatments (*P* < 0.05) within sampling time points.

Overall, relative RBC HSP70 levels were lower than those observed with liver HSP70 (Fig. 6). Similar to liver HSP70, a significant interaction (*F*_12, 160_ = 22.25, *P <* 0.001; Table 2) between thermal cycle and time was observed. RBC HSP70 was induced at the peak of the first heat cycle (*t* = 60 h) in all three-temperature treatments. At *t* = 60 h, RBC HSP70 in 22–27°C group was significantly higher than the other treatments (*P <* 0.001; Fig. 6). However, at the next sampling point (*t* = 72 h), relative RBC HSP70 was greater in the 18–27°C group than in the other temperature treatments. At the end of the experiment (*t* = 120 h), RBC HSP70 remained elevated in all thermal groups, but was significantly lower in the 20–27°C group compared to the 18–27°C and 22–27°C groups.

**Figure 7:**
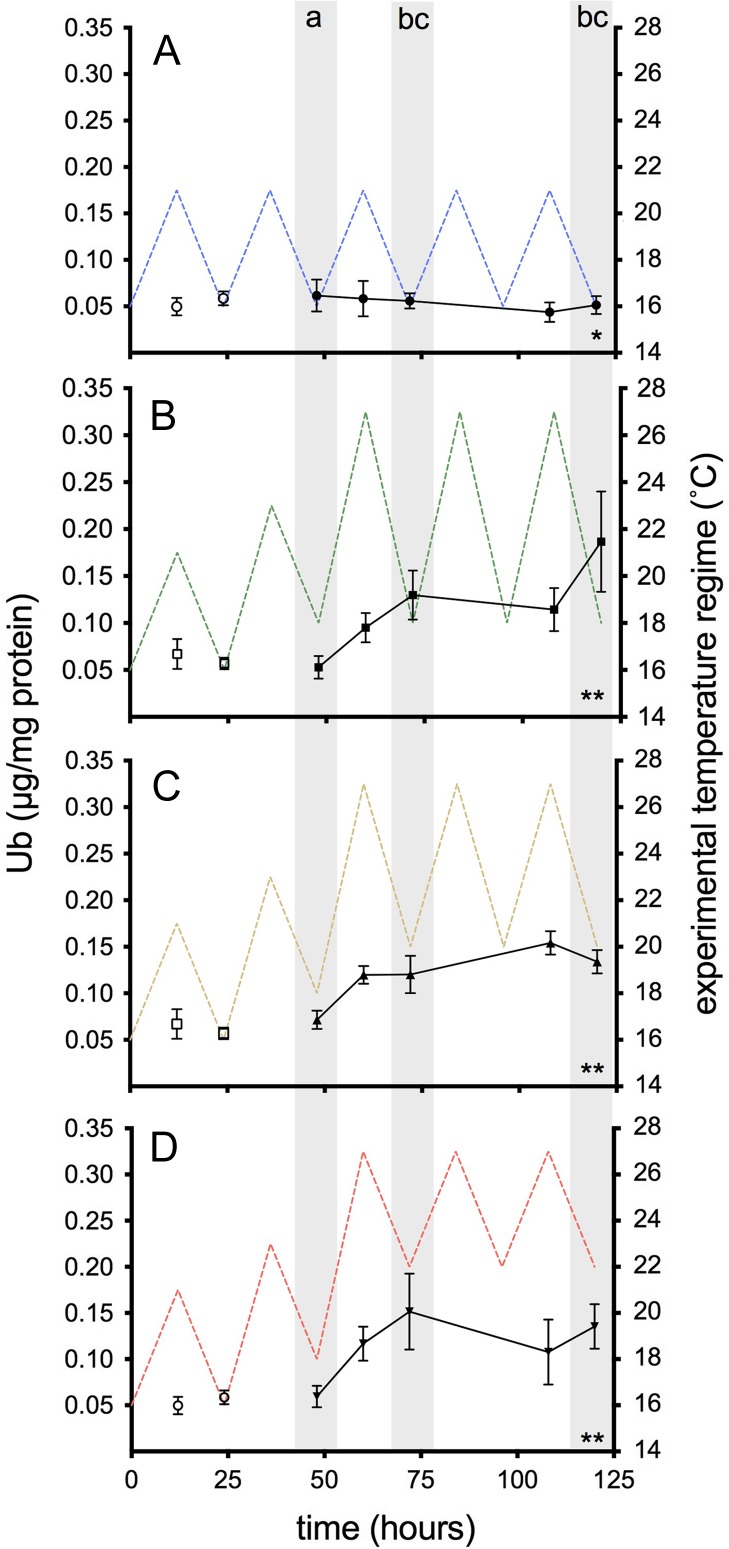
RBC Ub in juvenile salmon exposed to the following diel cycles: (**A**) 16–21°C only; or multi-day thermal stress (closed symbols; 60–120 h) of (**B**) 18–27°C; (**C**) 20–27°C; (**D**) 22–27°C. Points represent mean ± SEM (*n* = 4–9). Pre-thermal stress sampling (open symbols; *t* = 12 and 24 h) assessed the effect of ‘trial’ and was not included in the analysis. Asterisks indicate significant differences between treatments. Lettered shaded bars indicate significant differences between time points (*P* < 0.05).

As was the case with HSP70, RBC Ub levels were comparatively lower than in liver (Fig. 7). RBC Ub changed over time and between temperature treatments (*F*_4, 160_ = 4.78, *P* = 0.001; *F*_3, 160_ = 15.03, *P <* 0.001; Table 2). Similar to liver Ub, all three thermal groups had significantly greater RBC Ub than the control at 16–21°C (*P <* 0.001), but did not differ significantly from one another (*P* = 0.47–0.99). RBC Ub significantly increased at *t* = 72 h, after the peak of the first heat cycle (*P* = 0.007). Unlike the case in liver where Ub levels were no longer significantly different from pre-thermal exposure at 120 h (*P* = 0.91), RBC Ub remained significantly higher than the pre-thermal exposure (*t* = 48 h) after the final thermal cycle (*t* = 120 h; *P <* 0.001).

## Discussion

Environmental thermal patterning has been recognized as being intrinsically linked to fish physiology and performance (Helfman *et al*., 2009; Vasseur *et al*., 2014) and important for predicting the effects of climate change. We hypothesized that the nature of the thermal cycle, specifically nighttime temperature would dictate the physiological limits of juvenile Atlantic salmon. We found that our thermal cycles did initiate increases in CTMax and provided evidence of both cellular and metabolic stress. However, contrary to our expectation, we did not observe a correlation between the overall metabolic/cellular condition throughout a simulated heat event and the daily thermal minima. Thus, when fish are exposed to warming diel cycles approaching their critical temperature, we conclude that the diel thermal minima normally experienced by juvenile Atlantic salmon may not play a critical role in the ability of these fish to deal with extreme events. Notably, our experimental design, where we used a fixed maximum temperature, did not allow us to disentangle possible effects of the magnitude of diel thermal fluctuation from minimum nighttime temperatures. However, recent research from our group concluded that the nature of ecologically relevant diel thermal cycling (e.g. accumulated thermal exposure, magnitude, rate of change) did not significantly affect metabolism or the stress response in wild Atlantic salmon (Tunnah *et al*., 2017). Regardless, distinguishing between the importance of *T*_min_ and Δ*T* is an important direction for future research.

Exposure to ecologically relevant thermal cycles (16–21°C) increased acute thermal tolerance (CTMax) compared to fish held at a stable acclimation temperature (16°C). Furthermore, we determined that exposure to a single elevated high temperature pulse (27°C) increased CTMax values by ~1°C but neither diel *T*_min_ nor exposure to repeated diel pulses further elevated CTMax. Acclimation temperature has long been known to influence thermal tolerance in ectotherms (Beitinger *et al*., 2000); however, few studies have examined the effects of thermal cycles on CTMax (see Bennett and Beitinger, 1997; Currie *et al*., 2004; Fangue *et al*., 2011). In support of our findings, diel cycling did not affect CTMax in killifish (*Fundulus heteroclitus*) (Healy and Schulte, 2012) or the northern two-lined salamander (*Eurycea bislineata*) (Rutledge *et al*., 1987). In both cases, CTMax increased only when overall acclimation temperature was increased. Furthermore, Healy and Schulte (2012) suggest a complex association between variation in acute thermal tolerance and physiological (i.e. altered membrane fluidity) and environmental (i.e. photoperiod, hypoxia and time of day) processes. In nature, maximum temperature tolerance is poorly understood and thought to be a function of time, diel mean and Δ*T* (Wehrly *et al*., 2007) with periods of repeated sublethal stress capable of delaying mortality (Selong *et al*., 2001).

Our thermal cycles induced significant changes in blood lactate and liver glycogen, with minimal differences among thermal regimes. Alterations to the initial metabolic condition when an organism is exposed to increasing (LeBlanc *et al*., 2011) and diel thermal stress are often short lived (<24 h; Tunnah *et al*., 2017). In our study, transitory cyclic increases in both blood lactate and glucose occurred in the warm thermal cycles. The modest decreases in liver glycogen we observed with high temperature cycling may be indicative of rapid mobilization of free glucose to surrounding metabolically active tissues resulting in a loss of metabolic capacity (Viant *et al*., 2003). After 3 days of thermal cycling, no difference in liver glycogen was apparent among treatments suggesting a comparably high energetic demand in all our diel thermal cycles. Our results therefore suggest that salmon metabolically manage diel cycles in an equivalent manner, regardless of nighttime temperature, refuting the notion that warmer thermal minima are most strenuous (see DFO, 2012). Wilkie *et al*. (1997) exposed exercised Atlantic salmon to stable recovery temperatures and determined fish were able to recover to a pre-stress metabolic status faster at warmer temperatures (23°C vs. 12 and 18°C). There is some field evidence for the preference of warm recovery temperatures during periods of thermal stress. For example, juvenile salmonids have been observed dispersing from aggregations formed at cool water sources at nightfall despite temperatures remaining above the species’ thermal optima (Belchik *et al*., 2004; Corey *et al*., unpublished data). Although it is implied that this behavior is driven by a thermal cue, our results indicate that this response may not be directly associated with a metabolic or cellular advantage to a particular *T*_min_, but may be an effort to minimize the magnitude of temperature change experienced by the fish.

We know that temperatures >22°C will induce HSP70 in wild juvenile Atlantic salmon in the Miramichi River (Lund *et al*., 2002). Diel temperature stress induced an upregulation of six heat shock genes in redband trout (*Oncorhynchus mykiss gairdneri*) (Narum *et al*., 2013) and HSP70 in several tissues of Atlantic salmon (Tunnah *et al*., 2017). Given this information, the current temperature conditions of the Miramichi River, and regulations regarding recreational angling (closure when *T* ≥ 20°C for two consecutive nights; DFO, 2012), our prediction was that juveniles experiencing higher nighttime temperatures would display a higher magnitude heat shock response (HSR) than those experiencing a lower diel *T*_min_. Instead, our data indicated that exposure to diel, environmentally relevant, sub-lethal heat stress, had cellular level consequences but the nature of high temperature cycling (i.e. nighttime temperature) had minor effects on the HSR. Thus, the magnitude of the thermal stress, regardless of diel cycle, was consistent with the magnitude of the HSR. Assuming that thermally induced protein denaturation/damage triggers the HSR (Ananthan *et al*., 1986), our data suggest that protein damage is similar among our different diel cycles. In support of this, induction of Ub occurred at the peak of the first heat cycle and remained elevated throughout the heating event, regardless of thermal cycle, as was the case with HSP70. Tunnah *et al*. (2017) also demonstrated consistency of the HSR with Ub induction in thermally cycled Atlantic salmon.

Our measured physiological responses to thermal cycling were strikingly similar amongst the treatments and could be indicative of partial acclimation to increased temperatures caused by a slow ramping rate, or could suggest phenotypic plasticity to cope with thermal variability (Schulte *et al*., 2011; McBryan *et al*., 2013; Anttila *et al*., 2014). Metabolic stress in Atlantic salmon occurs at temperatures between 22°C and 24°C (Breau *et al*., 2011) demonstrating a limited ability to tolerate temperatures >28°C (Garside, 1973; Elliott, 1991), enduring 33°C only in acute circumstances (Elliott and Elliott, 1995). It is possible that genetic makeup and thermal history, specifically the frequency that these upper thermal thresholds are surpassed, may play a larger role in the metabolic and cellular status of juvenile salmon, while nighttime temperature may have little influence on a fish's ability to re-establish basal cellular and metabolic conditions. Wehrly *et al*. (2007) determined maximum temperature tolerance to be a function of time in two trout species, *Salvelinus fontinalis* and *Salmo trutta*, where prolonged warm periods negatively influenced upper thermal tolerance limits. Furthermore, in chronically warmed European perch (*Perca fluviatilis*), Sandblom *et al*. (2016) determined that, despite cardiorespiratory plasticity to deal with a warmer resting condition, the upper thermal limit remained relatively rigid in both cold and warm adapted fish, consequently reducing the available ‘thermal buffer’.

Understanding how animals cope with large thermal fluctuations is critical to safeguard species in a changing climate. It has been suggested that if the rate of evolutionary or plastic responses lag behind the rate of climate change, there could be local extinction due to limitations in physiological capacities compared to the environmental variation (Chown *et al*., 2010). Here, we investigated key markers of the stress response in Atlantic salmon to determine how fish physiology responded to distinct warming scenarios with different nighttime temperatures. If high nighttime temperatures resulted in physiological stress, we would expect that the warmest (22–27°C) treatment would elicit the most obvious signs of cellular and metabolic disturbance. However, this was not the case as different *T*_min_ thermal scenarios had little effect on our dependent variables. These findings lead us to reject the hypothesis that environmentally relevant nighttime temperature is a principal driver in the ability of Atlantic salmon to tolerate multi-day thermal events at, or near, critical temperatures. With future climate change scenarios predicting an increase in thermal extremes and increases in diel thermal minima, it is likely that physiologically important thresholds will be surpassed more frequently. Current regulations regarding recreational angling close the Miramichi River when *T* ≥ 20°C for two consecutive nights (DFO, 2012). Our results suggest that these regulations be revisited given that the overall effects of ‘warm nights’, at least within the temperature ranges tested here, do not appear to be a critical factor influencing Atlantic salmon. We do show that environmentally relevant diel thermal cycles up to 27°C are stressful for these fish; thus, management decisions should focus on ecologically grounded and relevant simulations of thermal stress and pay attention to maximum temperatures and Δ*T*. While turning down the temperature of the planet is not an option, an understanding of such biological responses to warming water temperatures will inform the design of effective habitat management strategies to ensure the availability of cool water refugia, protecting Atlantic salmon during these inevitable thermal challenges.
